# Anxiety and Depression in British Horseracing Stud and Stable Staff Following Occupational Injury

**DOI:** 10.3390/ani13213337

**Published:** 2023-10-26

**Authors:** Emma Davies, Sophie Liddiard, Will J. McConn-Palfreyman, John K. Parker, Lorna J. Cameron, Jane M. Williams

**Affiliations:** 1 Equine Department, Hartpury University, Gloucestershire GL19 3BE, UKlorna.cameron@hartpury.ac.uk (L.J.C.); jane.williams@hartpury.ac.uk (J.M.W.); 2 Sport Department, University of Stirling, Stirling FK9 4LA, UK; 3 Sport Department, Hartpury University, Gloucestershire GL19 3BE, UK; john.parker@hartpury.ac.uk

**Keywords:** mental health, racing grooms, retention, wellbeing, workforce, racehorse welfare

## Abstract

**Simple Summary:**

Employee mental health is a strategic initiative for global organizations and maintaining staff wellbeing is a key focus for British horseracing. Workforce wellbeing is increasingly linked to employee recruitment, retention, and productivity, challenges currently facing the horseracing sector. Improving staff wellbeing is paramount to maintaining high standards of equine welfare, ensuring the industry’s social license to operate is upheld. Research in horseracing has identified a range of factors influencing staff wellbeing; however, the role of injury in anxiety and depression scores is unknown for this population. Over two thirds of injured staff were experiencing anxiety and over half were experiencing depression. Anxiety and depression scores were related to employment status, working hours, and type of injury. Higher anxiety and depression scores were negatively associated with help-seeking and pain management behaviors during injury, as well as increased risk of using alcohol as a coping method, both for pain-relief at work and socially. The findings from this study may provide opportunities to influence mental health post-injury within horseracing, through the development of educational resources aimed at reducing stigma, improving mental health literacy, and developing industry-wide early screening protocols for mental health in injured staff.

**Abstract:**

Horseracing has identified several factors influencing staff wellbeing; however, the relationship between injury, anxiety, and depression is yet to be established. This study investigated anxiety and depression scores and their association to pain management, coping, and help-seeking behaviour in injured British horseracing staff. An online retrospective survey was completed by 175 participants, identifying injury prevalence, coping strategies, occupational risk factors, and Hospital Anxiety and Depression Scale (HADS) scores. Analysis identified 65.14% (*n* = 114) of staff reported anxiety scores above the threshold (≥8) and 59.52% (*n* = 104) of staff reported depression scores ≥8. Median anxiety and depression scores were higher for staff who viewed their employer as unhelpful (anxiety *p* = 0.001; depression *p* = 0.020). Heightened anxiety and depression were associated with an increased likelihood to use pain medication to manage at work, including non-steroidal anti-inflammatory drugs (NSAID’s), alcohol, nicotine, and prescription drugs (*p* < 0.05). Implications for staff wellbeing is evident; anxiety and depression risks are high following injury, which may influence help-seeking behaviour, perceived job security, and coping mechanisms. This paper suggests it is vital to continue to investigate poor mental health and injury in racing staff and the implications for equine welfare.

## 1. Introduction

Mental health is a key focus for global organisations, and there is an increasing social pressure for companies to identify how they are supporting mental health in their employees [1], including within British horseracing organisations [2,3]. Mental health can be defined as the “state of well-being in which the individual realizes his or her own abilities, can cope with the normal stresses of life, can work productively and fruitfully, and is able to make a contribution to his or her community” [4]. Concerns for racing staff mental health have already been reported; increased pressures due to a “relentless” fixture list [5,6,7], lack of work–life balance resulting in a reduced recovery time [5,6] and high rates of injury [8,9] have been identified as significant risk factors. An industry report suggested 72% of training yard staff experienced stress, anxiety, or depression in the prior 12 months [3]. Targeted support for employee and employer mental health is a strategic priority for the horseracing industry [2,10,11,12] and considerations such as working hours or the fixture list are under review by the sector [2,10]. Yet, despite this, there is little research considering the relationship between occupational injury and mental health in horseracing staff.

Occupations with high physical and emotional pressure often have perceived assumptions that participants are physically and mentally strong [13], resilient and self-sufficient [14], and accustomed to working through pain [15], which, whilst required for the demands of the job [14], becomes problematic for mental health when those assumptions are challenged, for example due to injury [16]. Horseracing is a physically, mentally, and emotionally demanding occupation [9,17], and multiple risk factors have recently been identified that may increase the risk of injury for horseracing staff [8,9]. There is a high prevalence of injury in horseracing, with staff self-reporting an average of 3.3 injuries per annum [8]. Industry data suggests over 50% of yards report more than 1 serious accident per year [18,19,20] and hospital visits were required in 28–71% of cases, highlighting the severity and nature of injury type is wide within the horseracing industry [19]. Whilst the horse is considered the primary causal factor, 40% of equine-related accidents are caused by human acts or omissions, supervision, organisational structure, or environment, with risk factors including mental or physical fatigue, injury, limited range of movement, flexibility, or function to perform tasks; or low morale [21]. Recent horseracing research noted the disregard for personal injury in stable staff [3,8] and a culture of presenteeism, turning up to work when injured or unwell, which could further increase the risk of accidents when working around horses to staff and the horses they are caring for [21]. These attitudes suggest an injury minimalization culture within horseracing [6,19] that needs to be challenged to ensure optimal health and wellbeing of the workforce. Employees who ignore their own health needs may experience higher levels of physical and mental stress, increasing the risk of mental ill-health and risk of re-injury [22].

There is a bi-directional relationship between mental health and injury [23], whereby poor mental health is a risk factor for injury incidence and complicates recovery outcomes, but injury is also a contributing factor for new incidences of mental illness [16]. Injury has been linked to increased incidence of depression [16,24] and anxiety [25] in several different populations. Depression is defined as a mood disorder, characterised by “pervasive low mood, loss of interest in usual activities and diminished ability to experience pleasure” [26]. Depressive episodes are categories based on the prevalence of a wide range of symptoms, including but not limited to reduction in energy, low mood states, tiredness, loss of interest or enjoyment, sleep disruption, or changes in appetite [27]. Depending upon the number and severity of the symptoms, a depressive episode may be specified as mild, moderate, or severe [27]. Injury research has investigated the full range of this definition, from temporary emotional disruption to diagnosable clinical depression [16,24]. Depression is a global public health issue, with approximately 280 million people experiencing depression [28] and is not uncommon amongst athletes [29], military service personnel [14], jockeys [30,31,32], trainers [33] and stable staff populations [3]. Research suggests, in 5–21% of cases, moderate to severe depression is seen post-injury, which can cause dysregulation of the immune system, harming tissue repair [24], and negatively influencing future injury risk [16]. Clinical depression has been shown to increase cortical activation in pain contexts, resulting in an increased experience of pain severity, which can alter adherence to rehabilitation and increase fear of movement [34,35]. This has previously been reported as a concern in older racing staff [2,20]. Increased incidence of depression post-injury has also been linked to decreased productivity in the workplace, absenteeism, presenteeism, and increasing healthcare costs in wider sectors [4]. Depression can also contribute to other issues following injury, such as sleep disturbance, adverse alcohol and substance misuse, and suicidal ideation [36]. Substance use can cause retardation of healing, with delays in cell migration and collagen production, resulting in a suboptimal recovery following injury [37], thus highlighting a need to monitor depression and associated coping mechanisms within injured populations.

Anxiety is a “physiological state characterised by cognitive, somatic, emotional, and behavioural components producing fear and worry” [26]. Often accompanied by somatic symptoms including elevated heart rates and shortness of breath, anxiety is a prominent consideration in injured athletes [38]. Anxiety can affect pain perception at the onset of injury; anxiety has a similar physiological response as pain, utilizing similar hormone and immunological functions, and is often classified as a potentiator of pain [39]. Heightened anxiety is correlated to an enhanced perception of pain due to a down-regulation of immune function resulting from an increase in stress hormones which interfere with pain modulation [37]. Pain at the onset of injury can result in avoidance behaviors, social isolation, and psychological distress [40]. Anxiety, independent from its impact on pain recognition, is also likely to alter someone’s recovery behaviors. Increased anxiety is correlated with reduced adherence to rehabilitation, usually linked to social isolation or fear of negative social evaluation, which can stem from a lack of confidence [37,41]. Due to this, anxiety is a vital factor in injury management that needs to be further understood in the horseracing sector.

Employee health and wellbeing are paramount to an industry’s successes: a healthy workforce is consistent with improved quality of work, optimal working environments, employee retention and recruitment, and the sustainable development of the industry [42]. For horseracing, employee health can also impact the quality of equine care provided, potentially influencing the health and welfare of the horse. Reduced concentration, physical fatigue and burnout resulting from injury, a stressful working environment, or reduced mental wellbeing can negatively impact task efficiency by affecting visual acuity, accuracy, and individual reaction time [43]. Slower reactions, or loss of focus around horses, could result in preventable injury to both parties, negatively affecting human and horse health [21]. There is a plethora of research suggesting physician mental health influences patient care [44], risk of medical errors [45], and patient mortality [46]. Whilst different in context, the role of racing grooms is that of ‘care’, and it is reasonable to assume that poor mental wellbeing in animal care professionals may reduce the quality of care provided to the animals under their care, comprising health and welfare [43], although currently very limited research exists to validate these claims. Animal welfare standards have been closely linked to occupational wellbeing and mental health in farmers [47], and animal care workers [43], suggesting poor employee mental health may result in suboptimal management and care of the horse, reinforcing the importance of a healthy workforce to not only safeguard racing staff but also to maintain high equine health and welfare standards within the sector. The aim of this study, therefore, was to investigate anxiety and depression scores in injured British horseracing stud and stable staff, and their association to pain management, coping strategies, and help-seeking behaviours. The objectives were, therefore, as follows: (1) Identify anxiety and depression scores in injured horseracing staff, and possible associated risk factors; (2) Determine the relationship between mental health, help-seeking behaviours, and injury attitudes; (3) Examine the association between mental health, pain management practices, and coping strategies.

## 2. Materials and Methods

### 2.1. Study Design

A descriptive, cross-sectional, retrospective online survey design was used in this study. Online surveys are an effective tool to interact with a more diverse respondent group and allow researchers to access larger sample sizes conveniently, and within COVID-19 restrictions (survey dated January–February 2021) [48]. Previous research has questioned the validity of retrospective data collection in reflecting on experiences of injury [49]; however, both racing and equestrian athletes have been found to recall injury experiences and reflect on both positive and negative emotions successfully [50,51], whilst Southwick et al. [52] suggests that memory recall is remarkably accurate for injury experiences. As horseracing staff are required to identify injuries within the yard accident book at the time of injury [10] this may allow for greater memory recall to facilitate the completion of the survey [49]. The survey was piloted using a purposeful sample (*n* = 10) of local horseracing staff.

### 2.2. Participants and Recruitment

Following institutional ethics approval by the Hartpury University Human Research Ethics Committee (approval number ETHICS2019-67) and informed consent, eligible British horseracing staff (*n* = 352) voluntarily provided anonymous online survey data. Inclusion criteria were horseracing staff over 18 years old and employed in the British horseracing industry for a minimum of 12 months in a horse-handling related role (i.e., stud grooms, racing grooms, assistant trainer, trainer, or work rider). The survey was open to all horseracing staff who met the above criteria, irrespective of whether they had experienced any injuries in the prior 12 months. Exclusion criteria included participants working in administrative roles, or those staff who were either not actively employed in the racing industry, or who had been employed for less than 12 months.

Recruitment was achieved through personal and organisational industry contacts, collaborating partners and social media groups/pages to recruit participants who meet inclusion/exclusion criteria [53]. Participants were not offered incentives to complete the survey, but industry organisation endorsement of the survey was provided through membership newsletters, social media pages, and websites to target staff eligible to participate. Engagement with surveys in recent years has been considered a strength of employees within the racing industry [54], and similar methods have been utilized to gain prior injury and mental health data from stable staff [3]. Potential respondent bias was minimized by utilizing a wide range of online sites to recruit participants, such as Facebook^®^, Twitter^®^, Instagram^®^, and through direct communication with the industry via organisations and member bodies [48].

### 2.3. Measures and Procedure

The online survey was conducted using Qualtrics CoreXM 2021^®^ survey software. Participants completed 24 closed and 2 open questions, which took approximately 11 min to complete. Questions were designed by the research team, informed by Speed and Anderson [7], Filby and Jackson [18], and Filby, Jackson and Turner [19], to investigate injury prevalence, risk factors, and injury reporting behaviours within horseracing staff, and covered four areas of significance: employee demographics, employment characteristics, injury characteristics, and injury management, including coping strategies. Justification for the inclusion of these areas is provided in Table 1 and a full copy of the questionnaire can be found as supplementary materials in Davies et al. [8]. Staff were asked to complete the Hospital Anxiety and Depression Scale (HADs), a 14-item self-report questionnaire comprising statements designed to detect anxiety and depression scores within participants [55]. The questionnaire consists of 14 statements measuring self-perceived feelings of two scales, anxiety (HADS-A, *n* = 7) and depression (HADS-D, *n* = 7), graded from 0–3, with a maximum possible score of 21 for each. Scores are tallied individually for both anxiety and depression, and according to the Pais-Riberio et al. [56] interpretation, the score 0–7 represents “normal”, the score 8–10 represents “mild”, the score 11–14 represents “moderate”, and 15–21 “severe”. The HADs is a validated questionnaire assessing anxiety and depression, with excellent internal consistency (0.8–0.83) [56,57] and has shown good agreement with other mental health questionnaires [58,59,60]. Expressed by Cronbach’s α coefficients, both HADS-A (α = 0.87) and HADS-D (α = 0.83) scales had excellent internal consistency in this study. Inter-item correlations for HADS scales were >0.3 (HADS-A) and >0.2 (HADS-D), meaning all items on each scale correlate well with the scale overall.

Participants were informed of their data protection rights and procedures for withdrawal before being asked to consent to the study; no identifiable data were collected. The survey was live from 7 January 2021, following an industry sector launch, and closed on 28 February 2021.

### 2.4. Data Analysis

Data were exported from QualtricsXM to Microsoft Excel (Office 365). Of the 352 participants who provided data for the survey [8], a total of 175 participants were included for analysis specific to this study. Data exploring wider aims, injury prevalence, and risk factors utilising all 352 participants is published by Davies et al., [8]. To meet this study’s aims, participants were required to have experienced an injury in the prior 12 months, removing 11.9% (*n* = 42) of participants from the original sample (*n* = 352), and must have provided completed answers to all required sections relevant to the aims of the present study, including the Hospital Anxiety and Depression Scale (HADS) questions.

For the remaining 175 participants, HADS statements (question 22) were tallied (HADS-A, statements 1–7; HADS-D statements 8–14) to identify HADS-A and HADS-D total scores as a scale variable, ranging from 0–21. In addition to the HADS scale data, tallied scores were then categorised into four groups: normal (0–7), mild (8–10), moderate (11–14), and severe (15–21) [56] as an additional ordinal grouping variable. Frequency analysis was used to assess anxiety and depression scores, injury prevalence, reporting behaviours and pain management practices. Data were analysed using IMB Statistical Product and Service Solutions (SPSS) software version 26; significance was set at *p* < 0.05 unless otherwise stated. All data met the assumptions as non-parametric data. Mann–Whitney U tests for difference (Bonferroni-corrected *p* < 0.003) were used to compare median anxiety and depression scores between staff reporting specific injury types to those who had not reported that injury type in the previous 12 months. Mann–Whitney U tests for difference also compared median anxiety and depression scores between genders and staff who used drugs and alcohol at work to those who did not. Kruskall–Wallis tests for difference compared median anxiety and depression scores between several categorical occupational variables including employment status, years in racing, working hours, perceptions of job control and job security, helpfulness of support networks, likelihood of injury reporting behaviours, as well as average weekly alcohol intake. Chi-squared tests for independence were used to identify associations between ordinal HADS-A and HADS-D categorical data and access to support services, pain management behaviours, and social drug-taking behaviours. Finally, Spearman’s correlations were used to identify linear relationships between anxiety and depression scores and age, reported as a continuous variable.

## 3. Results

### 3.1. Demographics

Data from a total of 175 participants were analysed in this study (Margin of Error ± 7% at 95% CI), with an average age of 34.3 years ± 10.64 years, and divided into twenty-nine (16.57%) males, one hundred forty-four (82.29%) females, and two (1.14%) who did not state their gender.

Most participants were working full-time in racing (*n* = 107, 61.14%), whilst a further thirty-seven (21.14%) staff worked part-time, twenty-four (13.72%) were self-employed, three (1.71%) staff were on long term sick leave, and four (2.29%) were currently not employed at the time of the survey. The largest single category of employees had worked in racing for between 1–5 years (*n* = 61, 34.6%), with 42.29% of participants working in the industry for more than 10 years (see Table 2).

A total of 711 injuries were reported for 175 staff in this survey. Staff reported between 1 and 12 injury types each (Mode = 4, Median = 4), with an average of 4.06 injuries/person in the prior 12 months.

The most common injuries reported were bruises, with 90.9% of staff reporting bruising in the last 12 months (*n* = 159), in addition to lower back pain (*n* = 106, 60.6%), muscle strain (*n* = 100, 57.1%), and upper back/neck pain (*n* = 67, 32.3%). A full breakdown of injuries can be seen in Table 3.

### 3.2. Prevalence of Anxiety & Depression in Injured Racing Staff

Of the 175 injured stud and stable staff who completed the HADS, 65.2% (*n* = 114) reported mild, moderate, or severe anxiety scores (cut off threshold ≥8), whilst 59.4% (*n* = 104) reported depression scores above the cut-off threshold of ≥8 (See Table 4). A significant positive relationship between HADS anxiety and HADS depression scores was recorded (r_s_ = 0.597, *p* < 0.001).

### 3.3. Factors Influencing Anxiety and Depression in Injured Racing Staff

#### 3.3.1. Demographics

No significant relationship between age and anxiety or depression or significant differences between genders were found for injured stable staff (*p* > 0.05). No significant differences between length of career (years in racing) and anxiety or depression were found in injured stable staff (*p* > 0.05).

#### 3.3.2. Employment Status

Significant differences in depression were found between the employment status of injured staff (H (4) = 11.686, *p* = 0.020). Staff working part-time reported significantly lower median depression (M =7) than full time staff (M = 9, *p* = 0.007) or director/limited company owners (M = 12, *p* = 0.008). No significant differences in anxiety were found between employment status in injured staff (*p* > 0.05). No significant differences were found in anxiety or depression for injured racing staff between different sectors of the racing industry (for example, flat racing, jump racing or stud) (*p* > 0.05).

#### 3.3.3. Average Daily Working Hours

Significant differences in anxiety (H (5) = 15.325, *p* = 0.009) and depression (H (5) = 16.190, *p* = 0.006) were reported by injured staff dependent on their average daily working hours. Injured staff working over 12 h/day reported increased median anxiety (M = 12.5), whilst those working 10–11 h/day reported the highest median depression (M = 11) compared to those working fewer hours.

#### 3.3.4. Injury Type

Median anxiety and depression were compared between those staff who reported a specific injury (injury event) and those who had not experienced that type of injury (non-event) in the prior 12 months. Staff who had experienced lower back pain, lower extremity fractures and muscle strain all reported higher median anxiety or depression compared to those who had not reported those specific injury types in the survey (*p* < 0.003) (Table 5). Higher median anxiety and depression were found in staff who reported that injury type (injury event) compared to staff who did not report that injury type (non-event) in all significant comparisons (Table 5).

### 3.4. Anxiety and Depression and Help-Seeking Behaviour and Injury Attitudes in Injured Horseracing Staff

Median anxiety scores differed significantly between staffs’ perceptions of how helpful they found support from other individuals, including for their children (H (5) = 13.162, *p* = 0.022), work friends (H (5) = 16.317, *p* = 0.006), and employers (H (5) = 21.147, *p* = 0.001). Furthermore, significant differences in depression were found between staff perception of employer helpfulness (H (5) = 13.350, *p* = 0.020). Staff who perceived their employer has more helpful reported lower anxiety and depression scores, whilst staff who perceived children and work friends as more helpful also reported lower anxiety scores. Higher median anxiety and depression scores were found for staff who perceived their support networks as unhelpful compared to those who felt support was helpful during recovery in all comparisons (Table 6).

Chi-squared analysis revealed no significant associations between HADS scores and whether staff were more likely to access either Racing Occupational Health Services (*p* > 0.05) or Racing Welfare (*p* > 0.05) during their injury. Anxiety and depression did not seem to be associated with help-seeking behaviours from racing service providers in injured horseracing staff.

No significant differences in anxiety or depression were found between the likelihood of staff to seek medical attention for all four injury types (fracture, other musculoskeletal injury, concussion, or other head injury) (*p* > 0.05). Significant differences in anxiety H (5) = 12.920, *p* = 0.024) and depression (H (5) = 13.106, *p* = 0.022) were found between rated likelihood to report concussion to an employer. Staff who were extremely likely to report concussion to their employer reported lower median anxiety (M = 8) and depression (M = 8) than those who were extremely unlikely to report (HADS-A M = 12; HADS-D M = 9) or were unsure (anxiety M = 12; depression M = 10). Furthermore, significant differences in depression (H (5) = 11.537, *p* = 0.042) were found between rated likelihood to report musculoskeletal injuries to an employer. Staff who were unsure whether to report a musculoskeletal injury to an employer reported the highest median depression (M = 10.5), compared to those who were extremely likely to report (M = 8, *p* = 0.010) and extremely unlikely to report (M = 6.5, *p* = 0.007). No significant differences in anxiety or depression were found between likelihood to report fractures or other head injuries (*p* > 0.05). Significant differences in anxiety were found between the likelihood of staff to take time-off following concussion (H (5) = 12.651, *p* = 0.027). Staff who were extremely likely to take time-off following concussion had lower median anxiety (M = 8) than those who would continue working (M = 12.5, *p* = 0.002). No significant differences in anxiety or depression were found for likelihood to take time off for any other injury types (fracture, other musculoskeletal injuries, or other head injuries) (*p* > 0.05).

### 3.5. Anxiety and Depression and Pain Management Practices in Injured Horseracing Staff

Injured staff who reported using no drugs to manage daily tasks at work had significantly lower reported anxiety (M = 7, *p* = 0.001) and depression (M = 6, *p* = 0.006) compared to those who reported using drugs as a coping method (HADS-A M = 10; HADS-D M = 9). Use of NSAIDs to manage daily tasks at work was significantly associated with anxiety scores (χ^2^ (3, 175) =11.718, *p* = 0.008). Staff using NSAIDs to manage at work were more likely to have higher anxiety scores. Use of nicotine (in all forms) to manage daily tasks at work was significantly associated to depression scores (χ^2^ (3, 175), =14.897, *p* = 0.002). In addition, those staff who used nicotine to manage at work reported higher median anxiety (M = 10.5, *p* = 0.025) and depression (M = 11.5, *p* = 0.001) compared to staff who did not use nicotine (HADS-A M = 9; HADS-D M = 8). Use of prescription drugs (with prescription) to manage daily tasks at work was significantly associated to both anxiety (χ^2^ (3, 175) = 13.050, *p* = 0.005) and depression (χ^2^ (3, 175) = 10.765, *p* = 0.013). Staff using prescribed drugs to manage at work were more likely to be experiencing higher levels of anxiety and depression. Injured staff who reported using alcohol to manage daily tasks at work reported significantly higher anxiety (M =11.5) compared to those who did not use alcohol as a coping method (M = 9; *p* = 0.011).

### 3.6. Anxiety and Depression and Social Risk-Taking Behaviour in Injured Horseracing Staff

No associations were found between social drug taking behaviours and anxiety or depression levels in injured staff (*p* > 0.05). No significant associations were found in anxiety or depression levels between betting frequency (*p* > 0.05). There were significant differences in depression when compared between weekly alcohol intake (H (4) = 11.986, *p* = 0.017). Median depression was highest in those who drink more than 15 units/week (M = 10) and those who do not drink (M = 10), compared to those who drank 11–15 units/week (M = 6.5). There were no significant differences in anxiety between weekly alcohol consumption (*p* > 0.05) in injured racing staff.

## 4. Discussion

This study identified high anxiety and depression scores in injured staff, which were associated with employment status, working hours, and type of injury experienced. Negative perceptions of social support were found in injured staff with higher median anxiety and depression scores, as well as an increased risk of using alcohol as a coping method both at work, and socially. Finally, injured racing staff with higher anxiety and depression scores reported a reduced likelihood to report injuries and take time-off in the future.

### 4.1. Prevalence of Anxiety & Depression in Injured Racing Staff and Influencing Factors

Injured racing staff reported high anxiety and depression scores; over two thirds of staff reported high anxiety scores, whilst over half experienced high depression scores (above cut off threshold ≥8). Previous research in horseracing supports the high prevalence of anxiety and depression, with 72% of training yard staff experiencing stress, anxiety, or depression in the previous 12 months [3]; however, this study is the first to identify anxiety and depression scores specifically in injured horseracing staff. Anxiety or depression can significantly affect injury recovery [68]; increased anxiety and depression may influence perceptions of pain [69] and can result in avoidance behaviors, social isolation, and increased psychological distress [40,70]. Furthermore, depression has been linked to increased absenteeism, decreased productivity at work, and increasing healthcare costs in wider sectors [4] and issues of poor horse welfare could arise if staff are not fully engaged in their daily tasks. Routine screening post-injury has been recommended for hospitalised patients to identify anxiety and mood disorders [71,72,73], and this study would recommend this screening process is adopted within British horseracing. Racing staff have previously reported a reduced likelihood of attending hospital following a serious incident [8], but there are strict requirements for accident reporting within training and stud yards which are adhered to [8,10]. For serious occupational injuries, injured staff should be directed to an online screening questionnaire, provided by the Racing Occupational Health Service. The screening would aim to identify early symptoms of anxiety and mood disturbances following injury and could signpost at-risk staff to additional support. There is a reticence to access support services and ask for help in horseracing despite the range of resources available [74]. The industry, therefore, has a duty of care to enforce a more targeted approach to identifying and supporting at-risk individuals whilst also increasing mental health literacy for its stakeholders.

Common mental disorders previously reported in the horseracing industry are proposed to be associated with the novel occupational stressors and pressures staff experience within the industry [8,30]. There were several risk factors associated with higher anxiety and depression scores in this study, including employment status, working hours and injury type. Injured business owners/limited company directors reported the highest depression scores in this study. In training yards, directors or owners are often also the trainer, a hands-on, high-demand role that is considered critical to the industry [6]. Trainers are responsible for the care of the horse, management of employees, relationships with customers and owners, and the running of the business [7], responsibilities that are greatly impacted by personal injury. There is a limited focus on the health and wellbeing of horseracing trainers, despite research to support that small to medium enterprise business owners or managers are at increased risk of depression and burnout [75] due to the high emotional labour [6,33]. King et al. [33] recently found 41% of trainers surveyed showed clinical signs of depression, and this study suggests this population is at greater risk of depression following injury than other staffing groups. Injury appears to be a key risk factor for depression in racehorse trainers and future research should investigate narrative injury experiences of racehorse trainers, and the interaction of injury and mental health in career satisfaction.

Injured staff working longer hours were more likely to report higher anxiety and depression scores. Staff reporting working more than 10–11 h/day in the current study were at increased risk of depression, whilst working more than 12 h/day was associated with increased anxiety scores. Longer working hours have previously been linked to increased risk of depression [76], anxiety [77], fatigue and work-related stress [78], as well as increased alcohol use and smoking [42]. The demands of horse care and additional pressure of current labour shortages [5,6,8] means recommendations to reduce working hours for stable staff as a method to decrease injury risk and poor mental health outcomes are often viewed by employers as unrealistic. The authors would encourage an industry review of working hours and their influence on occupational wellbeing, but also recommend that other preventative measures be implemented, including modifications to job requirements on their return to work following injury [8].

Finally, the type of injury experienced in the past 12 months was also linked to anxiety and depression scores reported by horseracing staff. Increased anxiety and depression were linked to several musculoskeletal injuries, including muscle strain and lower back pain. Musculoskeletal injury and back pain are commonly reported in racing staff [8,79] and most workers with recurrent musculoskeletal pain or discomfort continue to work, reporting minimal time loss [80] but reduced workplace productivity [81]. Magni et al. [82] found that chronic musculoskeletal pain was associated with increased feelings of depression compared to those who reported being pain-free, whilst higher rates of anxiety and depression following injury were associated with increased likelihood of experienced chronic pain [83]. Persistent pain may result in increased feelings of anxiety, with individuals reporting concerns for the meaning behind their symptoms or the future implications of their pain on work or life activities [84]. Chronic lower back pain has been found to increase the prevalence of anxiety and depression [85], which correlates with the findings of this study, where median anxiety and depression scores were higher for staff with lower back pain. Although anecdotally viewed as ‘part of the job’ by those working with horses, musculoskeletal injuries, chronic pain, and/or back pain exhibit a clear association with anxiety and depression that should be of primary concern for occupational health practitioners working within horseracing.

Staff also had increased depression scores when dealing with lower limb fractures. Lower extremity fractures can be considered as serious physical injuries [83], often requiring hospitalisation [86]. Hospitalisation following serious physical injury is considered a significant risk factor for depression [86] and anxiety [71]. Research suggests 28–42% of traumatic physical injury cases admitted to hospital showed depressive symptoms and mood disorders, whilst anxiety incidence is reported between 16–40% up to 12 months post-injury [72]. Recommendations for routine post-hospitalisation mental health screening following traumatic physical injury are strongly encouraged by existing researchers [72]. Given the higher median anxiety and depression seen for injuries typically associated with hospitalisation in this study, future research should consider the implementation of mental health screening through the Racing Welfare’s Racing Occupational Health Service for staff who have experienced a serious physical injury in the workplace.

Brain injuries are typically associated with increased likelihood of depression [72]; this study found higher anxiety scores in horseracing staff with suspected or diagnosed concussion, although these outcomes were not significant. The suspected interaction between neurological injury, recovery success rates, and the uncertainty around recovery timelines can reduce mood and create feelings of anxiety during rehabilitation [15]. Uncertainty has been shown to increase the level of psychosocial morbidity [87]. Intolerance of uncertainty, namely the difficulty of not knowing, is typically associated with depression but also with other mental health problems, including generalized anxiety disorder [88], which may explain the increased anxiety seen in this population.

### 4.2. Anxiety and Depression and Help-Seeking Behaviours and Injury Attitudes in Injured Horseracing Staff

Higher median anxiety and depression scores were found in injured staff who viewed their employer, children, and work friends as unhelpful to injury recovery and were significantly less likely to report future injuries, or take time-off, especially for concussion. Research in athlete and military populations suggests people are more likely to rely upon social support networks, such as friends, family, or colleagues following injury [13,14]. Amateur and elite riders have previously emphasised the benefits of talking about injury with “horsey people” and felt there was an increased understanding and empathy experienced within the equine community [51]. However, Tveito et al. [80] suggested that, in occupational settings, workers show concern for vocalising injury or pain for fear of annoying colleagues, which may explain the negative perceptions of colleagues as sources of support seen here. Stigma associated with perceptions of weakness, vulnerability, and incompetence is considered the principal barrier to accessing support [89], and internalising stigma can decrease a person’s sense of worth and self-esteem following injury [36,90]. Recent horseracing research suggests there may be a negative societal stigma associated with injury, mental health concerns, and help-seeking behaviour within the sector [3,8,74]. Negative perceptions of help-seeking could increase the risk of social isolation and loneliness in horseracing staff. Recently, loneliness in farmers, resulting from social isolation, has been attributed to decreased animal welfare standards, such as social interaction, injury or pain, and production health indictors [47]. Social isolation may unintentionally reduce horseracing staff’s ability to maintain higher equine care standards within their daily role, due to increased risk of distraction, reduced cognitive function, reduced physical functioning, and increased absenteeism. The subjective perception of mental health directly influences the likelihood to seek treatment and support [14]; therefore, it is important for the horseracing industry to develop targeted educational resources which aim to reduce the stigma around injury, mental health, and help-seeking behaviour, and increase mental health literacy across the sector [13,91] to support employee wellbeing and equine welfare. Current mental health and industry support resources, such as those provided by Racing Welfare, should aim to increase their visibility and accessibility, which may increase utilisation of existing services.

Injured racing staff have also previously reported negative perceptions of their employer during recovery; 41–44% of injured racing staff found their employer unhelpful [3,8]; however, results from this study suggest these perceptions may be partially explained by elevated anxiety and depression. Decreased support from colleagues and supervisors has been shown to increase the risk of depression [92] which could imply the relationships between perceptions of support, injury and mental health are more complex and warrant further investigation. The implications for this are concerning; a lack of confidence in reporting safety concerns or discussing injury with employers is considered a critical risk factor for accidents [21] which could suggest there is not only a concern for staff wellbeing if injury and mental health concerns are hid from employers but also for health and safety in the workplace and equine welfare. Previous research suggests there may be benefits in peer-led approaches to mental health education in non-trusting communities [14], such as horseracing [3,74], and the industry has already made significant investments into the subsidised training of staff for mental health first aid. Further training could specialise in injury-associated trauma, including training racing yard employees in Trauma Risk Management (TRiM) to increase the likelihood of peer-to-peer interaction following injury and provide opportunities for social support.

### 4.3. Anxiety and Depression and Pain Management Practices in Injured Horseracing Staff

When experiencing increased anxiety and depression, injured staff in this study were more likely to use drugs and alcohol to manage daily tasks at work, specifically increasing their use of NSAID’s, nicotine, and prescription drugs for pain management. The use of analgesics as a tool to comply with presenteeism is seen in several vocations, including sporting athletes [93]. The practice of using analgesics to maintain performance, improve recovery, and reduce the impact of injury has become a socially accepted practice in sport, often encouraged by peers and coaches as “routine” [93], and has also been reported in both horseracing [3,8] and equestrian research [94,95,96] as common practice for maintaining work and performance standards. However, the influence of drugs and alcohol in the workplace is considered a significant risk factor in occupational accident analysis [21], thus suggesting that, whilst commonplace in horse sport, there may be increased risk of accidents to those consistently using increased pain management strategies.

### 4.4. Anxiety and Depression and Social Risk-Taking Behaviour in Injured Horseracing Staff

Substance use and risk-taking behaviour are considered common coping mechanisms for a range of issues, including traumatic circumstances, social or environmental crises, work-related stress, and personal injury [97,98]. Use of alcohol and drugs as coping mechanisms following injury is widely reported, in both occupational and sporting environments [37,99], and is often accompanied by increased rates of anxiety and depression [97]. Whilst no associations between betting, social drug use, anxiety, or depression were found in the current study, injured staff with higher anxiety scores were more likely to be drinking socially, whilst staff consuming more than 15 units/week were at increased risk of depression. The comorbidity seen between alcohol and anxiety [100] is often attributed to the use of alcohol to self-medicate, whereby increased amounts of alcohol are reported to decrease symptoms of anxiety and depression [98]. For patients following surgery, higher levels of anxiety and depression were risk factors for increased reliance on alcohol [37]. Furthermore, in military professionals, alcohol is considered the most common coping method post-injury [101] akin to the findings of the current study. Alcohol negatively influences the body’s ability to heal from injury, resulting in sub-optimal recovery [37,100] and increasing the risk of reinjury or long-term impairment.

However, average alcohol intake reported in this study should be considered with caution. The current study obtained data during January and February 2021, requesting injury data from the prior 12 months, during which there were several periods of national lockdown due to COVID-19. Global crises, such as the COVID-19 pandemic, have been shown to increase alcohol intake in the general population, as well as decrease overall mental health and wellbeing [97,102]. Approximately half of surveyed participants changed their alcohol intake during the pandemic, with 24.6% of people increasing their weekly alcohol consumption [98]. Change in typical alcohol consumption, either increasing or decreasing, has been associated with heightened risk of anxiety and depression, as those experiencing reduced mental wellbeing are more likely to change their habits [98,103]. This study did not identify if alcohol or substance use changed following injury experiences and given the complexities of the global climate at the time of data collection, caution should be taken in inferring that increased alcohol consumption reported here is a direct result of either injury or heightened anxiety or depression.

## 5. Limitations

There are several limitations to consider within this study. The use of self-reporting to identify staff’s pain management regime, social drug use, average alcohol consumption, and betting behaviours is open to bias and may likely represent an underestimation of social risk-taking activities [99]. The use of the HADS scale, whilst validated for use in injured populations [56,57,58], is limited to only assessing the severity of self-reported symptoms of anxiety and depression; thus, it may not represent a true picture of mental health following injury. Furthermore, this study does not identify pre-injury anxiety or depression scores and, thus, cannot infer cause and effect in the relationships between injury, anxiety and depression in this population. Data were gathered between January and February 2021 and, thus, data are inclusive of the impact of the COVID-19 pandemic. Whilst occupational demands remained consistent for racing staff during the pandemic, staff concerns over mental health were identified during the first COVID-19 lockdown [104]. Wider research acknowledges the significant impact of the pandemic on measures of mental health in numerous populations [97,105,106] and, thus, caution should be taken when interpretting these results beyond the pandemic landscape.

The online sample which allows access to a wide population of staff in a rapid manner may introduce self-selection biases [107]. This may result in responses being skewed to include only staff whose injuries have caused significant impact, so should be treated with caution and may not be truly representative of the general population of staff within horseracing. Industry data from 2018 reported over 6700 registered racing employees working on licensed training yards and a further 3500 staff working on registered stud farms [108], although no data exists determining the proportion of staff experiencing injury. Although the survey was promoted throughout and by the industry, a smaller percentage than expected of the eligible population responded. Injury reporting is generally low within this population and is a concern already being discussed at length in Davies et al. [8] and Davies et al. [9]. Injury minimalization culture [109] is commonplace in other vocations, such as the military, where 49–58% of musculoskeletal injuries are unreported. Racing research shows that staff tend to conform to organisational expectations suppressing and regulating emotional responses that do not typify the “tough” characteristics expected of racing staff when injured or in pain [6,20]. Cassidy [17] suggests the organisational culture of horseracing may encourage employees to act, think, and feel in adherence to cultural norms, and staff are taught to meet these cultural norms at the outset of their career. Additionally, horseracing yards were traditionally male domains [110] propagating the “tough” retoric that emphasised physical strength and resilience [111]. Whilst the ratio of male: female staff is changing, with 70% of staff in yards identifying as female [112], this study represents only 16.57% of male participants, which is not representative of the current horseracing workforce. The low male sample size could be explained by the influence of gender on help-seeking behaviours, which typically sees men less likely to seek help due to hyper-masculine ideologies, such as a reported indifference to pain [111]. Male racing employee engagement with mental health research should be closely monitored moving forwards. The results of this study suggest that, in horseracing, these cultural and gender norms may include being seen to be tough, working whilst in pain, masking injuries, or disguising emotions to avoid, at best, losing respect in the workplace or, at worst, their job. This perceived organisational culture is likely to influence honest reporting of injuries, hindering further research in the area. 

## 6. Recommendations and Future Research

Given the findings of this study, several recommendations and future directions are proposed. Further research should continue to explore the bi-directional relationship between injury and mental health in horseracing staff, considering the long-term implications for increased anxiety and depression in injured staff. In addition, this study has highlighted an increased risk of injury-associated anxiety and depression in trainers (e.g., directors and company owners). Further research should investigate the narrative experiences of trainers following personal injury and the effects this may have on mental health and wellbeing, management of their business, and their intentions to remain in the sector. Due to heightened anxiety and depression seen in this population, this study also proposes that further evaluation of the interrelationship between human and horse health and wellbeing is warranted, to generate an increased understanding of whether employee mental health may disrupt the synergy of the dynamics of racehorse care, to enable the racing industry to fulfil its social license to operate. Recommendations from this study include the following:

1. The development and dissemination of educational resources aimed at reducing injury stigma and increasing mental health literacy to be made available for horseracing staff across the sector;
2. Increased visibility of Racing Welfare’s provision and services within the wider sector to maximize recognition and utilization of available services;
3. An industry-wide protocol for studs and training yards to refer injured staff (reported in accident book) for an online mental health screening questionnaire, implemented by Racing Occupational Health Service, which would highlight at-risk employees who could be contacted for early intervention;
4. Additional industry training to support peer-to-peer interactions at work, focusing on injury-associated trauma and mental health.

## 7. Conclusions

The occupational health of stable staff is a key priority for the racing industry, and it is becoming increasingly more important to consider the nature of injury and mental health for stable staff, in line with the industry’s strategic priorities. The present study is the first to identify anxiety and depression scores in injured horseracing staff, risk factors, and to identify their associations with help-seeking behaviour, pain management, and coping strategies. Injured staff reported high anxiety and depression scores, which were influenced by employment status, working hours, and type of injury experienced. Anxiety and depression negatively influenced help-seeking and pain management behaviours during injury, as well as increased the risk of using alcohol as a coping method, both for pain-relief at work and socially. Higher levels of anxiety and depression were also found in staff who had a reduced likelihood to report concussion and take time-off in future. As the occupational wellbeing of animal caregivers has previously been reported to affect animal welfare standards in other sectors (veterinary, farming, and rescue centres), further research is recommended to investigate the implications of racing staff mental health on quality of equine care in horseracing and subsequent racehorse health and welfare. The findings from this study may be used to develop industry-wide early screening protocols for mental health assessment in the injured staff of the horseracing industry.

## Figures and Tables

**Table 1:** Questionnaire topic areas.

Topic Area	Key Focus	Justification
Demographics	Age, biological sex, years in industry, geographical location (region)	Discrepancies in injury prevalence between age groups and gender seen in previous research [7,18,19,61]. Age and gender are predictors for poor mental health [62]. Demographics also impact risk of substance misuse [63].
Employment characteristics	Job type, full or part time contract, hours, pay, job control	Job characteristics and limited job control is a key factor in work-based injury and stress [64].
Injury Characteristics	Injury type, incidence, experience of injury	Injury causation and situational context are factors that may affect cognitive appraisal of the injury [65], changing emotional responses, and rehabilitation/coping behaviors [66].
Injury management and attitudes to injury (including coping mechanisms)	Approaches to injury management (personal and professional), pain management practices, support networks	Under-reporting is an anecdotal concern for the racing industry. Institutional habitus and expectations of ‘toughness’ seen in racing staff [8,67].
Social coping behaviour	Self-reported social drug taking behaviour, average alcohol consumption, gambling behaviours	There is a relationship between types of substance abuse and depression and anxiety [63]. McConn-Palfreyman et al. [3] suggest that drugs are used as a coping mechanism to de-stress.
Hospital Anxiety and Depression Scale (HADs)	Anxiety and Depression Scores	There are significant mental health concerns within the industry [3]. Injury is considered a predominant risk factor in the diagnosis of anxiety and depression [68].

**Table 2:** Number of years working in horseracing.

Length of Career in Racing	N	Percentage Total (%)
1–5 years	61	34.6%
6–10 years	40	22.9%
11–15 years	24	13.8%
16–20 years	18	10.4%
21–25 years	13	7.4%
26 + years	19	10.9%

**Table 3:** Workplace injuries reported by horseracing staff in the last 12 months.

Injuries	N	Percentage Sample ** (%)
Bruises	159	90.9%
Lower back pain (lumbar)	106	60.6%
Muscle strain	100	57.1%
Upper back or neck pain (cervical/thoracic)	67	32.3%
Concussion (suspected)	43	24.6%
Lacerations	42	24%
Tendon/Ligament damage	42	24%
Sprained ankle	26	14.9%
Fractures—arms or hand	23	13.1%
Rib bruising or rib fractures	19	10.9%
Sprained wrist	16	9.1%
Concussion (diagnosed by clinician)	13	7.4%
Fractures—leg or foot	13	7.4%
Nerve damage	12	6.9%
Dislocation e.g., shoulder or knee	11	6.3%
Other *	10	5.7%
Other head injuries	5	2.9%
Fractures—skull	2	1.1%
Fractures—spine	2	1.1%

* Other included pelvic or hip fractures or internal injuries. ** These values represent a % of the total participants (*n* = 175) who reported a given injury, as participants were able to report more than one injury type within the survey.

**Table 4:** Reported Anxiety and Depression in Injured Racing Staff.

	Mean ± SD	Range	Normal (Scores 0–7)	Mild (Scores 8–10)	Moderate (Scores 11–14)	Severe (Scores 15–21)
Anxiety	9.51 ± 3.80	2–18	61 (34.9%)	46 (26.3%)	47 (26.9%)	21 (12%)
Depression	8.51 ± 3.16	2–18	71 (40.6%)	60 (34.3%)	35 (20%)	9 (5.1%)

**Table 5:** Median Anxiety (HADS-A) and Depression (HADS-D) per injury grouped by staff who reported the injury type (injury event) and staff who did not report the injury type (non-event).

Injury Type	Anxiety Median (Injury Event)	Anxiety Median (Non-Event)	Depression Median (Injury Event)	Depression Median (Non-Event)
Bruises	10	7	9	7
Concussion (diagnosed by clinician)	12	9	10	8
Concussion (suspected)	10	9	9	8
Dislocation e.g., shoulder or knee	9	10	9	8
Fractures—arms or hand	9	9	10	8
Fractures—leg or foot	11	9	12 **	8 **
Lacerations	10	9	9	8
Lower back pain (lumbar)	10 *	7 *	9	7
Muscle strain	10 *	8 *	9	8
Nerve damage	13	9	10.5	8
Other	10.5	9	7.5	8
Other head injuries	11	9	9	8
Rib bruising or rib fractures	10	9	11	8
Sprained ankle	12	9	10	8
Sprained wrist	12	9	10	8
Tendon/Ligament damage	9	9	9	8
Upper back or neck pain (cervical/thoracic)	10	8.5	9	8

* Significant differences for HADS-A Bonferroni-corrected (*p* < 0.003). ** Significant differences for HADS-D Bonferroni-corrected (*p* < 0.003).

**Table 6:** Median anxiety (HADS-A) and depression (HADS-D) per source of support between those who found their support extremely helpful during injury and those who found support extremely unhelpful.

Support Network	Anxiety Median (Extremely Helpful)	Anxiety Median (Extremely Unhelpful)	Depression Median (Extremely Helpful)	Depression Median (Extremely Unhelpful)
Employer	7 *	12 *	7 **	9.5 **
Children	12.5 *	15 *	10	10
Work Friends	7 *	15 *	7	10
Spouse	9	11	8	7
Parents	10	14	8	10
Family	10	14	8	10

* Significant differences for HADS-A (*p* < 0.05). ** Significant differences for HADS-D (*p* < 0.05).

## Data Availability

Data is confidential and, thus, not publicly available for access.
